# Engaging people with long-term health conditions in a community-based physical activity initiative: a qualitative follow-up study evaluating the *parkrun* PROVE project

**DOI:** 10.1186/s13102-021-00351-8

**Published:** 2021-10-10

**Authors:** Helen Quirk, Steve Haake

**Affiliations:** 1 School of Health and Related Research (ScHARR), The University of Sheffield, 30 Regent Street, Sheffield, S1 4DA UK; 2 Advanced Wellbeing Research Centre, Sheffield Hallam University, Sheffield, UK

**Keywords:** Physical activity, Volunteering, Evaluation, *parkrun*, Disability, Long-term health condition, Qualitative, Community

## Abstract

**Background:**

The “*parkrun*: running or volunteering for everyone” (PROVE) project was an example of a community-based physical activity and volunteering initiative for people living with long-term health conditions in England. The 3 year project involved appointing volunteer Outreach Ambassadors whose role was to promote *parkrun* to people living with long-term health conditions through various outreach activities. This qualitative study aimed to understand the experience of delivering the project from the perspective of volunteer Outreach Ambassadors and the PROVE Project Manager.

**Methods:**

The PROVE Project Manager and ten PROVE Outreach Ambassadors across nine health condition groups were interviewed by the researcher (asthma, blood pressure, deaf and hard of hearing, dementia, diabetes, endometriosis, heart conditions, learning disabilities and/or autism, and obesity). Interview transcripts were analysed using thematic analysis.

**Results:**

Four themes and nine sub-themes were generated. The participants highlighted challenges in measuring the project’s success and bringing about meaningful and lasting change, and reflected on the value of the project as a learning opportunity. Despite some successes, it was thought that the project had limited reach outside of the existing *parkrun* community. The Outreach Ambassadors reflected on their experiences in the role and the skills required, finding it rewarding and highlighting the importance of networking and forming connections with key stakeholders. The findings are discussed in comparison to interviews conducted with the Outreach Ambassadors 12 months earlier.

**Conclusions:**

This study provides evidence to support the public health potential of *parkrun* though targeted initiatives such as the PROVE project and provides a critical reflection on what worked and what did not work when delivering the project. The findings have relevance for organisations wishing to implement similar outreach initiatives using a volunteer workforce, including recommendations for resource management, communication, leadership, fostering volunteer autonomy and defining and capturing success.

## Background

The evidence supporting the role of physical activity in the management of health conditions such as coronary heart disease, cancer, diabetes, asthma, multiple sclerosis, severe mental illness, arthritis and dementia is compelling [1–8], but significant challenges can prevent people from being more active [9]. The most common barriers often relate to the symptoms experienced (e.g. pain, breathlessness), but importantly, other challenges include lack of understanding about the suitability of activities and the perceived or real lack of access [9]. In England, government strategies advocate increased participation in physical activity among inactive and socially marginalised groups [10]. Strategies such as *Uniting the Movement* [11] and campaigns such as *We Are Undefeatable* [12] have a vision of providing opportunities for everyone to be physically active, regardless of age, background or ability. They have tended to follow the principle of ‘proportionate universalism’ whereby interventions, policies and campaigns are universal (i.e., not targeted at single groups), but are implemented with intensity (effort) and scale (reach) proportionate to the level of social need and/or disadvantage [13]. Identifying and addressing the various barriers to implementation and participation by certain underrepresented population groups, such as those living with long-term health conditions and disabilities, is crucial for these strategies to work [14].

Many groups in society face barriers to physical activity participation and thus are often underrepresented, including people living with long-term health conditions and disabilities. Long-term health conditions (referred to here as ‘health conditions’, for brevity) are conditions that require ongoing management for a number of years. People living with health conditions are nearly twice as likely to be inactive compared to those without a condition and this figure increases with the number of conditions reported [15]. This highlights a problem with a universal approach to physical activity promotion that can actually increase health-related inequality [16]. Health-related inequality sees people from certain groups such as those with health conditions or disabilities being less likely to be active and derive the health and social benefits from a physically active lifestyle. The emergence of public health policies, strategies and recommendations facilitates the development and implementation of practices that can reduce discrimination and create opportunities for inclusive physical activity participation and better health outcomes among this population [17]. Yet to address this adequately, more needs to be done to understand *how* physical activity providers and community initiatives can promote inclusivity for all. To help provide guidance for organisations delivering community-based health initiatives, we examine this in the context of *parkrun*.

### Background to parkrun

*parkrun* (www.parkrun.com) is a community-based initiative offering weekly activity opportunities to communities across the world. It delivers 5 kilometre (km) events for adults and children in public spaces [18]. Over 170,000 people walk, jog, run and volunteer at a 5 km *parkrun* across more than 800 locations in the UK every weekend. Worldwide, these figures are around 300,000 people per week across 23 countries. Its growth has been described as 'organic' and demand-driven, as it was largely promoted via word-of-mouth and events were launched in 'every community that wants one' [19]. *parkrun* has been recommended in the World Health Organization's Global Action Plan on Physical Activity 2018–2030 as an intervention which provides free, accessible, whole-community opportunities for physical activity (including volunteering) [20].

Research evidence suggests *parkrun* can positively impact health and wellbeing [21]. However, an unintended consequence of its organic growth is the risk that certain groups and communities are less well represented in the *parkrun* population. For example, research suggests that the majority of *parkrun* participants in England and the UK tend to be of white ethnicity [16, 22, 23] and of higher socioeconomic status [24, 25]. Research has not yet looked closely at *parkrun* participation rates by people living with health conditions.

There is a need for community-based approaches that complement the health provision provided in primary and secondary care and can be scaled up to have population impact [26, 27]. To maximise the health and wellbeing impact to their events, in 2016 *parkrun* started to take a more proactive approach to engaging people from all ages, backgrounds and abilities. This meant taking steps to better understand the barriers to participation and developing solutions to removing them. This paper focuses on the PROVE (*parkrun*: running or volunteering for everyone) project, launched in England by *parkrun* in 2016. It was a three-year project to promote participation in *parkrun* by people living with health conditions.

### Background to the PROVE project

The ambition of the PROVE project was to encourage more people living with health conditions to participate in *parkrun*. It was implemented by *parkrun* in England between 2016 and 2019, overseen by a contracted Project Manager. The PROVE project has been described in detail in [28] and its components are outlined in Table 1. The project utilised a volunteer infrastructure akin to that used by *parkrun* globally. In total, 35 volunteer Outreach Ambassadors were appointed to design and implement outreach activities targeted at those living with health conditions. Outreach Ambassadors were required to have a specialist interest in the health conditions being targeted in the PROVE project, such as personal experience of the condition or as a health professional. Outreach Ambassadors were recruited by *parkrun* from within the *parkrun* community. Outreach activities aimed to encourage participation by those people and groups who might not ordinarily take part (e.g., due to lack of awareness of access barriers) and who might benefit doing so. The outreach activities were not identified prior to the start of the project, but were instead developed by the Outreach Ambassadors as part of the dynamic implementation process itself.

**Table 1:** Overview of the PROVE project outreach activities

PROVE project activity	Description of the activity	Outputs*
Identifying the health condition groups	Health condition groups were identified by the PROVE Project Manager on a convenience basis, based on need and demand	The PROVE project targeted 13 health condition groups: Asthma; Blood pressure; Cardiovascular; Deaf and hard of hearing; Dementia; Diabetes; Endometriosis; Learning disabilities and/or autism; Obesity; Arthritis and musculoskeletal; Multiple Sclerosis; Cerebral Palsy; Mental Health
Appointing and managing Outreach Ambassadors	The PROVE project was led by the Project Manager who oversaw a volunteer network of Outreach Ambassadors. Applicants underwent an interview prior to being appointed as Outreach Ambassadors. Outreach Ambassadors were appointed to design and implement outreach activities targeted at those living with health conditions	35 Outreach Ambassadors were appointed which included 1–6 Outreach Ambassadors for each health condition group
Accessibility guidelines for *parkrun* event teams	Outreach Ambassadors wrote accessibility guidelines for *parkrun* event teams to raise awareness about the health conditions being targeted	20 Accessibility Guidelines were written and published on a Wiki web page used by *parkrun* event organisers
Facebook groups	Facebook groups were set up as an open forum for discussion and social supported related to *parkrun*. Groups were overseen by Outreach Ambassadors who monitored the content and prompted conversation	12 Facebook groups were established representing all condition groups except Cerebral Palsy (due to it being one of the latter conditions to be targeted in PROVE). These Facebook groups collectively had 5,907 members. The group with the highest membership was asthma with 2,153 members
Blog articles	Outreach Ambassadors published blogs covering stories of *parkrun* participants living with health conditions. Blog articles were published on the *parkrun* website and shared in weekly *parkrun* newsletters	42 blog articles were published over the course of the PROVE project, resulting in around ~ 70,000 hits (clicks/hits), according to *parkrun* data insights
Engaging with charities and/or advocacy groups	The Outreach Ambassadors reached out to advocacy groups (e.g., charities) related to their health condition with information about parkrun and the PROVE project in attempt to reach wider audiences	Evidence from the PROVE Project Manager suggested that Outreach Ambassadors engaged with at least 30 different advocacy groups over the course of the PROVE project. Examples of engagement included presentations at the Diabetes UK Professional Conference 2018 and a national GP social prescribing conference, podcast interviews and newspaper articles
Promotional materials / resources	Where a need was identified, Outreach Ambassadors developed publicity/promotional materials for use by *parkrun* and the Facebook groups	Easy read guides were produced by the Outreach Ambassador for learning disabilities and/or autism: https://blog.parkrun.com/uk/2018/09/28/autism-and-learning-disabilities-the-power-of-information/ A promotional video was produced by the Outreach Ambassador for asthma: https://biteable.com/watch/parkrun-for-people-with-asthma-1856387/ These were shared via social media
Adaptations to *parkrun* events	Outreach Ambassadors identified barriers to participation in *parkrun* events that might be experienced or perceived by people with health conditions and suggested simple changes that would address any barriers	Outreach Ambassadors for the deaf and hard of hearing group identified that the pre-event announcements were inaccessible to some people with hearing impairments. In response, *parkrun* introduced a "Sign Language Support" volunteer role responsible for communicating the pre-*parkrun* briefing in British Sign Language. Data insights showed that this role was undertaken 1,129 times in the UK since it was introduced in April 2017 at 137 *parkrun* venues
Strava club groups	Strava is a mobile App and website for runners/walkers to connect to each other. Outreach Ambassadors created Strava 'Clubs' for the health condition they represented. This was a social support platform for *parkrun* participants with health conditions to log activities, connect and support each other	11 Strava groups were established, attracting over 800 members
Volunteer takeover events	Outreach Ambassadors organised volunteer takeover events. Volunteer takeover events are when a local club, organisation or group undertake the majority of volunteer roles at one *parkrun* event. The aim was to raise awareness amongst the *parkrun* community of *parkrun* participants with health conditions, and promote the value of volunteering as a means to enhance health and wellbeing	Around 27 volunteer takeover events—with a focus on different health conditions—took place across England over the course of the PROVE project

*All reported numbers correct as of the end of the evaluation period in March 2019

*parkrun* chose to focus on the following health conditions; arthritis/musculoskeletal, asthma, blood pressure, cerebral palsy, deaf and hard of hearing, dementia, diabetes, endometriosis, learning disabilities and/or autism, mental health, multiple sclerosis, and obesity. Each health condition was represented by at least one Outreach Ambassador, with some conditions being represented by up to six Outreach Ambassadors (e.g., diabetes). A previous paper reported findings from interviews conducted with Outreach Ambassadors within two months of them being recruited [28]. The current study is a follow-up with the same Outreach Ambassadors, seeking their reflections on what worked, what did not work and what was learned through delivering the PROVE project.

### Aim

This qualitative study aims to understand the experience of delivering the PROVE project from the perspective of the Outreach Ambassadors and the PROVE Project Manager and provide guidance for organisations wanting to implement similar outreach initiatives.

## Methods

### Participants

Participants were the PROVE Outreach Ambassadors and the PROVE Project Manager. Outreach Ambassadors in England representing the following nine health condition groups were invited for interview: asthma, blood pressure conditions, deaf and hard of hearing, dementia (including Alzheimer’s Disease), diabetes, endometriosis, heart conditions, learning disabilities and/or autism, obesity. Outreach Ambassadors for multiple sclerosis, mental health, cerebral palsy and arthritis/musculoskeletal conditions were not approached for a follow-up interview since they had not been in position for 12 months (they were appointed to the PROVE project later than the other Outreach Ambassadors).

The PROVE Project Manager and ten PROVE Outreach Ambassadors across nine health condition groups (asthma, blood pressure, deaf and hard of hearing, dementia, diabetes, endometriosis, heart conditions, learning disabilities and/or autism, and obesity) were involved in this research. Demographic details of the participants (gender, age, occupation etc.) were not collected to protect individual confidentiality.

### Procedure

Following ethical approval from the local research ethics committee and support from the *parkrun* Research Board, recruitment occurred between March 2018 and November 2018 using a purposeful sampling procedure. Only those Outreach Ambassadors who had participated in a previous interview were approached with an invite to be interviewed again. The interview findings reported here are for the interviews conducted in most cases around 12 months after each participant’s first interview, reported in a previous paper [28]. Outreach Ambassadors were appointed at different times depending on when the first interview took place, meaning the 12-month interviews were conducted between April 2018 and November 2018.

Ten Outreach Ambassadors who were already known to the lead researcher due to their participation in the first interview 12 months earlier, and who had given permission to be contacted again by the researcher, were contacted via email with an invite to interview along with a participant information sheet. Those willing to be interviewed were asked to sign an electronic consent form prior to the interview being arranged. Ten Outreach Ambassadors gave consent to be interviewed, as did the PROVE Project Manager.

A semi-structured topic guide was developed by the lead researcher in advance to ensure consistency in the topics covered across interviews. Interview questions included: *Has the project achieved what it set out to do? How has the project been implemented? What has worked well? What has not worked? What impact has the project had? What have been the biggest challenges? What does it mean to be a PROVE Outreach Ambassador?*

All interviews were conducted by the lead researcher, who is trained and experienced in qualitative research. The lead researcher is a female researcher working in the field of psychology, public health and physical activity promotion. She has no personal experience of living with a health condition but does have personal interest and experience of *parkrun* as a runner, walker and volunteer, which improved ability to build rapport in a more ‘natural’ interaction with interviewees. At the time of writing this paper, she is Deputy Chair of the *parkrun* Research Board, an independent research board hosted at an academic institution in the UK. From an epistemological point, it is important to acknowledge the interrelationship between the researcher, the data collected, the research participants and *parkrun*, as the organisation being researched. During data collection and analysis, it was important for the researcher to make conscious efforts to be critically reflexive about her personal and professional experience with *parkrun* to avoid imposing personal perspectives on the study, but instead use this to connect the data with her own ongoing experiences within the research context. This approach has been used by authors of other *parkrun* research studies [29].

Interviews with nine Outreach Ambassadors were undertaken on the telephone due to the geographical spread of interviewees. One interview was conducted in writing (electronic) via email due to the participant being unable to be interviewed by telephone. The same technique used in the first interview with this participant, as reported in our previous paper [28]. The interview with the PROVE Project Manager was conducted in person. Interviews were recorded with a digital sound recorder. Interview length ranged from 21 to 69 min and the mean interview duration was 39 min.

### Data analysis

Audio recordings of the interviews were transcribed by an external transcription company. Interview data were analysed thematically by the lead researcher who conducted the interviews. This allowed for greater immersion, familiarisation and recall of conversations. The analysis followed an iterative process of thematic analysis similar to that described as ‘codebook thematic analysis’ by Braun and Clarke [30]. This approach was appropriate to ensure that the data answered the pre-determined research questions and satisfied the purpose of the evaluation (i.e., fitted within pre-existing domains such as ‘what has worked well’, ‘what’s not worked’ and ‘what has the impact been’) [30]. The analysis approach involved reading and re-reading the transcripts systematically (transcript by transcript), generating initial codes (identifying important sections of the transcripts, attaching labels to them and thinking about how they relate to the rest of the data), grouping similar codes into topic areas that formed themes and sub-themes (bringing together ideas that are relevant to the research question as topic themes and identifying broader patterns of meaning), reviewing and refining themes and sub-themes, naming themes and finally writing up the findings (weaving together the data extracts with analytical narrative). This was not a sequential process, but iterative, with the researcher moving backwards and forwards between the phases.

Analysis was primarily deductive, with the researcher being led by topics of interest to answer the research question for the purpose of satisfying the aims of the evaluation of the PROVE project. That said, the researcher was open for themes being driven by the data and sought to be active and reflexive in the generation of themes (inductive analysis). Verbatim quotes were used to illustrate themes and sub-themes. The software NVivo (version 11) was used to facilitate the organisation of the data.

One researcher was involved in the process of coding and theming the data, but care was taken to enhance the rigour of the research. The researcher discussed the coding and themes with the wider research team and the PROVE Project Manager (peer debriefing; [31]), who offered alternative interpretations of the data. The interview transcripts and reporting of the findings were shared with the participants (member checking). The researcher was aware of potential biases that would influence the interpretation of the data including; her previous personal and professional experience of *parkrun*, her beliefs around physical activity, exercise and health and her professional relationship with *parkrun* staff and the PROVE Project Manager. The peer debriefing encouraged the researcher to reflect on her predispositions and interpretations of the data. Finally, the presentation of findings offers thick descriptions and verbatim quotes for transparency and to enable the reader to understand and interpret findings. Outreach Ambassador details have been removed from verbatim quotes to retain confidentiality.

The consolidated criteria for reporting qualitative research (COREQ) checklist was used as guidance in reporting the study [32].

## Results

Eleven people were interviewed, i.e., ten Outreach Ambassadors and one Project Manager. Thematic analysis resulted in the development of four themes and nine sub-themes relating to the reflections on the project’s ambition, thoughts about its implementation, perceptions of impact and feelings about being an Outreach Ambassador. These findings are presented in detail below and supported with verbatim quotes.

### Theme 1: Defining success and remaining realistic

Participants shared thoughts on the progress that had been made towards achieving the PROVE project’s ambition to encourage more people living with health conditions to participate in *parkrun*. This theme captures the responses.

#### Sub-theme 1a: What does success look like?

Participants all agreed that the main purpose of the PROVE project was to encourage more people living with health conditions in England to participate in *parkrun*. When asked to explain how that could be achieved, participants described needing to raise awareness of *parkrun* among people with health conditions in the general population and to ensure that *parkrun* is perceived as accessible to them, for example:

I’m looking to reach out into the community of people living with [health condition] and try to help them understand that *parkrun* is accessible and is there for them and it’s not just all about running, jogging and walking for fit and healthy people but that people with [health condition] will be made to feel welcome and that we are there to support them. (008, Outreach Ambassador)

Identifying and ‘lowering’ barriers to entry was suggested as a way of improving accessibility:

It's my role to help support, kind of help widen the appeal of *parkrun* and make sure it’s as inclusive as possible and that we’re doing what we can to help *parkrun* or help people with [health condition] feel that they could go to *parkrun* and feel included, and kind of raising awareness of the kind of challenges that having [health condition] might mean for taking part in *parkrun*. (016, Outreach Ambassador)

Outreach Ambassadors were less clear about how the project’s ambition was going to be quantified or measured. Some Outreach Ambassadors were unsure what constituted a successful outcome, for example:

When we spoke a year ago I said I didn’t know what success looked like and I think I still feel like that. Is success getting 10 more people with [health condition] to *parkrun* or is it 10,000 more people? (010, Outreach Ambassador).

#### Sub-theme 1b: Remaining realistic about the project’s potential impact

The Outreach Ambassadors and the Project Manger felt it was important to remain realistic about the potential for the PROVE project to bring about meaningful and lasting change in the proportion of people with health conditions participating in *parkrun*. For example, one Outreach Ambassador suggested that what they had achieved through the PROVE project was, ‘just not as earth shattering as maybe we first thought we were going to be’ (003, Outreach Ambassador). Similarly, another Outreach Ambassador said:

I guess maybe it’s a bit like with any project you kind of go into it with really grand ideas and then when you sort of figure out the kind of actual realities you sort of realise you have to do things on a smaller scale or make your goals a bit smaller (016, Outreach Ambassador)

### Theme 2: Project implementation and management

Participants reflected on how the PROVE project had been implemented, including sharing their insights on what worked, what did not work and why.

#### Sub-theme 2a: Embracing the learning process

Progress towards the project’s ambition was believed to be slower than anticipated. One Outreach Ambassador referred to the progress as ‘organic’, inferring that the project had developed slowly and naturally. Similarly, another Outreach Ambassador referred to progress as ‘baby steps’ and ‘work in progress’ (003, Outreach Ambassador). Among some Outreach Ambassadors there was tones of disappointment about the progress made, for example:

I think we have made some progress. I’m a little bit disappointed in the amount of progress we’ve made. I think we could have done more. Some of that is the initial lessons you learn during the first year of anything like this. (011, Outreach Ambassador).

Despite some disappointment with the progress made, it was clear that the PROVE Outreach Ambassadors appreciated the importance of learning along the way and building upon successes and failures. They reflected positively on the learning process, ‘Like with most things you sort of learn through doing it. It’s really hard to know how I’d do things different’ (016, Outreach Ambassador). One Outreach Ambassador acknowledged that slow progress can be beneficial, ‘If [we] had pushed it forward quicker we might have made some mistakes.’ (014, Outreach Ambassador).

#### Sub-theme 2b: Working within the boundaries of a volunteer workforce

The Project Manager and Outreach Ambassadors reflected on the implications of the PROVE project utilising a volunteer network for its delivery. The Project Manager, who had been commissioned by *parkrun* to project manage the project, compared working with a volunteer organisation to that of a staff organisation:

I think one of the learnings across a lot of this has just been that there’s only so much - working with a volunteer organisation is very different from working with a staff organisation - there’s only so much you can reasonably expect volunteers to do in terms of commitments. (002, Project Manager)

Outreach Ambassadors shared the belief that there is a limit to what volunteers can be expected to do due to other commitments and time demands. The Outreach Ambassadors seemed comfortable doing as much or as little as their personal time allowed, and seemed to value having autonomy, as described by one Outreach Ambassador:

[volunteers] should feel as if they do as much as they think is right, and do the things that they think are right, and things that they can manage. Because as much as we want to get the message out there, everyone’s a volunteer. (004, Outreach Ambassador)

#### Sub-theme 2c: The importance of project management

Participants reflected on the role of the Project Manager to oversee the implementation of the project and coordination of the volunteer Outreach Ambassadors. The Project Manager described the challenge of giving the Outreach Ambassadors autonomy whilst needing to be directive with guidance and instructions and 'hands-on' with final decision-making. For example, the following quote from the Project Manager captures the experience of 'striking a balance':

[the Project Manager role] really should be like gatekeeping and overseeing, but I think one of the learnings is that it strays into the 'hands-on' as well. It’s just been difficult over the last 12 months to get that balance between, and it’s always difficult, sometimes it’s easier to move things forward to just do it yourself. And it’s just about striking a balance about where you should dive in and do it yourself, and where it’s worth accepting that things are going to be maybe a bit delayed. That if you get the Outreach Ambassadors to do things, there’s that balance between wanting to empower them and give them their freedom, and being aware that we need to just get stuff done. (002, Project Manager)

The Outreach Ambassadors believed the Project Manager had been important, both strategically with project guidance and personally with support, for example:

I can’t imagine how this PROVE project could possibly have gone ahead without that kind of coordinating role and overseeing role. And I think [he/she] is particularly, [he/she]'s got particular skills. Like [he/she]'s really diplomatic but very clear with people. He/she holds you to account in the nicest possible way, you don’t even realise it’s happened until afterwards. He/she seems to grasp all the different nuances of the different conditions really well, so I think he/she’s quite amazing. (014, Outreach Ambassador)

There was generally a positive review of the project management, but some Outreach Ambassadors felt that the PROVE project was working in a silo and felt disconnected from other *parkrun* initiatives. A number of Outreach Ambassadors saw synergy between the PROVE project and the '*parkrun* practice' initiative (where healthcare practitioners signpost patients and practice staff to *parkrun* events), but lacked clarity on how to align the two initiatives together, for example:

[The initiatives] seem to be done in a very disconnected way. There’s one person working on the *parkrun* practice, and then the PROVE project seems to work in isolation. But in practice there’s a huge amount of overlap… and it's competing for the [organisation’s] resource and communications bandwidth. (011, Outreach Ambassador)

### Theme 3: Capturing impact

This theme captures the perceived impact of the PROVE project.

#### Sub-theme 3a: Questioning the scope of the PROVE project’s reach and engagement

The Outreach Ambassadors and the Project Manager spoke about the project's reach and engagement within and beyond the existing *parkrun* community. They described having received a positive response to the PROVE project and a general sense of support from the *parkrun* community, for example describing the response from the *parkrun* community as ‘overwhelmingly positive’ (002, Project Manager). Some Outreach Ambassadors believed that the PROVE project made people within the *parkrun* community more aware of people who have health conditions at *parkrun*. Some Outreach Ambassadors believed that it enabled conversations about health conditions, and opened doors to new opportunities for *parkrun*, for example:

It’s just been an opportunity to start conversations, and it’s given us that legitimacy to be able to say well, “why don’t you come along to the *parkrun* on a Saturday morning and we can help you to keep life as normal as possible” really ... So there’s a lot more communication, a lot more conversations going around, in my opinion anyway (003, Outreach Ambassadors).

I think with all these things it’s the drip, drip, drip effect. I think what PROVE is doing is harnessing lots of good things that are already going on, and nudging and pushing them forward, and bringing them to attention. (014, Outreach Ambassador)

Many of the activities implemented as part of the PROVE project (outlined in Table 1) facilitated the Outreach Ambassador’s ability to engage with existing *parkrun* participants. For example, the Facebook groups, blogs and the Accessibility Guidelines were said to have been received positively by the *parkrun* community. They were believed to help raise awareness of health conditions, shift the narrative, provide an opportunity to understand barriers to participation and what support people with health conditions might need. The challenge the Outreach Ambassadors had was in being able to measure the reach and impact the activities were having.

The Outreach Ambassadors and Project Manager also questioned the level of engagement they were getting beyond the *parkrun* community. One Outreach Ambassador cautioned, ‘I mean it had a good reaction, but as I say, we were largely speaking to the converted’ (011, Outreach Ambassador). This idea of the PROVE project 'speaking to the converted' was a belief shared by many Outreach Ambassadors, for example:

How do we reach people with [health condition] who don’t know about *parkrun*? We could try and reach them directly, but we need a huge budget. Trying to get other people to get our message out there is challenging. (010, Outreach Ambassador)

The Project Manager shared the opinion that the scope of the project’s reach was sometimes limited by communication challenges. The Project Manager described *parkrun* communication channels as like layers of an onion; from *parkrun* HQ at the centre of the onion and moving outwards to the *parkrun* core volunteer team, the *parkrun* community and the final layer being key stakeholders outside of the *parkrun* community that provide the links to members of the general population. Participants described challenges in reach and communication at all layers, for example, at the *parkrun* community level:

I’ve been to two or three [*parkrun* events] where I’ve just, different bits of the country where I’ve just gone up to the person on that day, the event run director, and said oh hi, just to say hi, I’m an Outreach Ambassador for PROVE. And they haven’t known about the PROVE project. (014, Outreach Ambassador)

The Project Manager described a communication challenge at the final layer of the onion:

A constant struggle is trying to get channels outside of *parkrun*. We’ve put loads of effort into different charities …. Even like the national bodies for various disability sports. And it just feels like you’re really, it just feels like you’re really struggling to get any engagement. And I don’t know whether that’s just because people like, they’ve got so much other stuff going on. It doesn’t feel like it’s a lack of goodwill engaging with these charities. When you get a response people are supportive and so on, but then you’ll agree something that’s going to get done, never gets done (P002, Project Manager)

Outreach Ambassadors felt that the success of the PROVE project depended on not only reaching wider audiences with messages, but about engaging and getting buy-in from those who can promote *parkrun* to wide audiences, such as healthcare professionals and other key stakeholders, for example:

Unless we get a buy-in from GPs (General Practitioners) and from community resources, you know, such as community groups etc., we can only do so much. We can bombard people, we can put posters out, we can put all sorts of fliers out, but we also need that recommendation, almost that qualification from healthcare professionals really (003, Outreach Ambassador)

Yet some Outreach Ambassadors described difficulties trying to form connections with advocacy groups, which were perceived as the gateway to the broader community of people living with health conditions. The Outreach Ambassadors described difficulty knowing who to speak to or how to reach them. Where interactions with advocacy groups did occur, it was facilitated by Outreach Ambassadors having an existing link or connection to the organisation.

#### Sub-theme 3b: Reliance upon anecdotal evidence of success

A common discussion point was the absence of measurable objectives and how this limited the ability to assess the impact of the PROVE project activities implemented. Most Outreach Ambassadors believed the primary desired outcome was to increase the numbers of people with health conditions participating in *parkrun*, but felt there was little quantifiable evidence to show that this was being achieved. *parkrun* does not routinely capture health condition data from its participants, making any quantifiable change difficult to capture in the evaluation of the PROVE project. Instead, the Outreach Ambassadors and Project Manager used anecdotal evidence and success stories, as demonstrated in the quotes below:

I don’t think we’ve got a very accurate or effective way of measuring [impact]… and in the absence of [evidence] almost comes down to an article of faith that’s showing we must be moving the dial a bit. And if we accept that it isn’t going to give us hard and fast evidence, then we’re into more of the qualitative [evidence] (002, Project Manager)

I think it’s really difficult. I mean I think we’re getting really positive stories on Facebook now. Would that have happened anyway? Who knows? I think a lot of it’s so hard to quantify, like the impact. When you read those stories it’s people doing, saying things like as a result of *parkrun* I’ve now done a 10k, or I’ve now gone out and run by myself (014, Outreach Ambassador)

Many people have benefitted. There are lots of examples of success stories. We hear and see examples all the time of people becoming involved in *parkrun* that didn’t think they could due to their health issues. Also as well as new people, I hear and see examples of people who were already going to *parkrun* but were missing out on the full experience, for example Sign Language Support now being available at many events on a very regular basis has improved things for them (005, Outreach Ambassador)

### Theme 4: What it takes to be a PROVE project outreach ambassador

Outreach Ambassadors reflected on their experience of being a PROVE project Outreach Ambassador.

#### Sub-theme 4a: Qualities and skills required

The Outreach Ambassadors were asked to describe their role and the characteristics of a successful Outreach Ambassador. The qualities defined were broadly similar to those described by Outreach Ambassadors in interviews 12 months earlier (e.g., communication skills, knowledge of the condition, empathy), with some additional qualities being valued 12 months later, like 'being good with social media', 'perseverance', 'tenacity', 'persuasiveness' and 'job flexibility' mentioned as important. Another key quality mentioned by Outreach Ambassadors was the ability to network and form connections with key stakeholders e.g., ‘[someone who] understands the need to make links with strategic organisations and across. So for us it’s across the voluntary, health services, social services…So they have to be strategic as well as doers’ (014, Outreach Ambassador).

Having existing links with stakeholders and an awareness of sources of support was believed to be helpful. It was common for Outreach Ambassadors to describe their role as important for signposting people to other services and organisations:

Even though it’s not necessarily part of our role to offer advice, if you can point people in the right direction at least, then I think we’ve probably done something useful. (004, Outreach Ambassador)

Our role as Outreach Ambassador isn’t to do things on the ground, I see it very much as facilitating and enabling and supporting, and getting people to think laterally and upwards and downwards about what they might do (014, Outreach Ambassador)

#### Sub-theme 4b: A proud and privileged position

All Outreach Ambassadors interviewed spoke positively about their experience as a PROVE Outreach Ambassador. There was a sense of pride and privilege. Many felt rewarded by the sense of ‘changing a few people's lives, really helping actual people’ (016, Outreach Ambassador). Value was placed on there being a team of Outreach Ambassadors bringing a range of skills and expertise to the role. Having more than one Outreach Ambassador was believed to be useful when other responsibilities took priority (e.g., personal life circumstances). Though working in teams brought about the challenge of living in different locations, making face-to-face meetings and decision-making difficult. One Outreach Ambassador explained:

I have found that decisions have been made and not involved everybody, and I’ve found that quite difficult to get my head around really. But I understand that sometimes you just have to go with it, sometimes because of the position that [the Project Manager] holds he/she obviously has the final say, and I appreciate that sometimes he/she just has to make that decision based on the information that he/she’s got. It’s not necessarily a criticism; it’s just obviously with the three of us being so far flung over the UK it can be difficult to meet at times (003, Outreach Ambassador)

There was a strong sense that the Outreach Ambassador role was considered a privileged position, ‘It becomes something you’re kind of quite proud of rather than a chore’ (015, Outreach Ambassador). It was common for Outreach Ambassadors to strive to want to achieve more. There was a strong sense of 'unfinished business' and a hunger to continue the work done to date, for example:

I wouldn’t say I’ve achieved everything that I wanted to do. It’s one of those things, it’s like moving the goalposts all the time isn’t it? Because of the way that [health condition] has such a negative impact on people, and on their quality of life, I don’t think my aspirations will ever be fully achieved…It’s just work in progress all the time (003, Outreach Ambassador)

## Discussion

This research interviewed *parkrun* volunteer Outreach Ambassadors and the PROVE Project Manager to explore their experience of delivering the PROVE project. The findings will be compared with our previous paper reporting findings from the interviews that took place with Outreach Ambassadors 12 months earlier [28].

There was agreement among Outreach Ambassadors that the objective of the PROVE project was to encourage more people with health conditions to participate in *parkrun* by raising awareness and removing barriers to participation. Outreach Ambassadors expressed the need to remain realistic about the potential for the PROVE project to bring about the desired change, which was consistent with earlier findings from the Outreach Ambassadors [28]. Follow-up interviews revealed that initial expectations about what the PROVE project could achieve within the three-year project were perhaps too high with respect to the resource available. This has been described elsewhere in the implementation of public health interventions as the, “delicate interplay between the ideal and the realistic” (31, p 19). There was also some uncertainty around what ‘change’ meant for the PROVE project and the definition of success. The challenge faced in capturing change raises the broader issue experienced in the evaluation of community-based programmes. Communities are complex and require multi-component and adaptable interventions that make traditional evaluation and monitoring tools ill-suited [33]. Alternative evaluation approaches are available, many of which attempt to address the shortfalls of traditional evaluation methods. Systems approaches [34], Realist Evaluation [35] and techniques such as ‘ripple effect mapping’ [33] have the benefit of being able to capture unintended as well as intended outcomes in complex programmes. Such approaches could be beneficial for mapping the various implementation barriers and might be more appropriate for evaluating the processes underlying initiatives like the PROVE project that have a number of attributes, are dynamic and seek to capture change beyond the intended objectives or outcomes.

The PROVE project utilised a volunteer workforce to design and deliver the programme of work. This approach is similar to that used by *parkrun* globally [18], which is praised for being a sustainable model of delivery [16]. One challenge for the PROVE Project Manager was with finding balance between giving the volunteers autonomy whilst providing structure and direction. Paradoxical leadership refers to two contrasting leadership styles being used simultaneously; “participative leadership aims at giving volunteers a sense of autonomy over their work and involving them in decision-making processes, directive leadership aims at providing them with clear goals and instructions on how to execute their tasks” (36, p 97). Although this is said to be a desirable approach for fostering positive volunteer engagement [37], the PROVE project demonstrated that this has challenges in practice and can slow down progress.

Yet despite these challenges, the Outreach Ambassadors found their role rewarding, felt a sense of pride and strived to do more, which are all characteristics of volunteer engagement [37, 38]. It would be worthwhile for further research to explore the conditions (e.g., leadership styles) that fosters engagement in *parkrun*’s volunteer infrastructure. Such research would help community organisations better understand the complex nature of engaging and retaining a volunteer workforce [37].

The perceived impact of the PROVE project was discussed in terms of reach and engagement. In earlier interviews, Outreach Ambassadors had perceived the PROVE project as a vehicle through which to promote *parkrun* to communities of people with health conditions [28]. The use of community networks is advocated as a means of supporting people living with health conditions to engage in physical activity [26]. Evidence shows that community-based approaches have the benefit of fostering socially supportive environments that can improve access and promote socialisation and engagement among people living with health conditions [26, 39]. For the PROVE project, having this community-based approach being driven by and leveraged by the community was reflected on positively [28]. However, in follow-up interviews, participants reflected that engagement occurred mainly within the existing *parkrun* community, rather than reaching out to the wider population. Thus, the PROVE project demonstrated that it may be easier to engage with those already in the *parkrun* system compared to reaching out and drawing in new participants, which may require greater resource, level of influence and expertise. In the earlier interviews, Outreach Ambassadors were optimistic that engaging with key advocacy groups would be a fruitful way of reaching wider audiences [28]. However, this was difficult for the Outreach Ambassadors to achieve unless existing links with external organisations were already established. Relationship building may need a strategic focus with a designated member of staff or Outreach Ambassador to broker and nurture partnerships.

This study identified some of the qualities deemed to be important for the fulfilment of the Outreach Ambassador role. Patience, persuasiveness, perseverance, strategy and job flexibility were suggested as important qualities for Outreach Ambassadors—in addition to the characteristics described in earlier interviews (communication skills, knowledge of the condition, teamwork and empathy [28]. These are consistent with findings from the volunteering literature [37].

The findings of this study may guide other organisations wishing to implement and evaluate community physical activity initiatives for people living with health conditions using a volunteer workforce. These can be summarised as follows:

1. It is important to be realistic about what can be achieved with the time and resource available;
2. Communication plans need to firstly identify the intended audience—including what relationships might already exist—and secondly outline plans to reach the target audience;
3. Paradoxical leadership may be required to manage a volunteer workforce, but can be challenging in practice;
4. Volunteers appreciate feeling valued, having autonomy and feeling well-connected with the organisation;
5. There must be a clear definition of success and outcomes of interest with various methods of measuring change, including capturing intended and unintended outcomes.

The findings should be interpreted in light of the following methodological considerations. The findings represent the views of self-selected Outreach Ambassadors and therefore people who are highly engaged with and supportive of *parkrun* and/or the PROVE project. Thus, readers should be aware of the potential self-selection bias of the data and be aware that the findings do not necessarily reflect the views of people with health conditions. Another limitation of this study is that the same researcher, who is a *parkrun* participant, collected and analysed the data so the findings should be interpreted with potential bias in mind. Furthermore, given that the researcher undertook peer debriefing with the PROVE Project Manager, this introduced a further potential for bias. Though the researcher did seek numerous ways to enhance the trustworthiness of the data (as described in the Methods).

## Conclusions

This study used *parkrun*’s PROVE project to explore how community-based initiatives can engage with people living with health condition who might not ordinarily participate. The PROVE project aimed to encourage more people living with health conditions to participate in *parkrun* and was delivered by a network of volunteer Outreach Ambassadors. This qualitative study aimed to understand the experience of delivering the project from the perspective of volunteer Outreach Ambassadors and the PROVE Project Manager. The findings provided recommendations for other organisations wishing to implement similar initiatives using a volunteer workforce. These were resource management, defining success, communication, leadership, and volunteer autonomy.

## Data Availability

The data sets generated and analysed during the current study are not publicly available due to the sensitive and identifiable nature of our qualitative data, but further details are available from the corresponding author on reasonable request.

## Abbreviation

PROVE: parkrun: running or volunteering for everyone
